# CD36 knockdown attenuates pressure overload-induced cardiac injury by preventing lipotoxicity and improving myocardial energy metabolism

**DOI:** 10.7150/ijms.107224

**Published:** 2025-02-18

**Authors:** Jing Geng, Xiaoliang Zhang, Ying Wang, Dong Guo, Panpan Liu, Siying Pu, Xue Yang, Qi Liang, Pan Chang, Tao Li, Lang Hu, Yanjie Guo

**Affiliations:** 1 The College of Life Science, Northwest University, Xi'an, Shaanxi, China.; 2 Department of Cardiology, Tangdu Hospital, Airforce Medical University, Xi'an, Shaanxi, China.; 3 Department of Cardiology, No.901 Hospital of PLA, Hefei, 230031, China.; 4 Department of Cardiology, Xi'an International Medical Center Hospital, Northwest University, Xi'an, Shaanxi, China.; 5 Department of Cardiology, The Second Affiliated Hospital of Xi'an Medical College, Xi'an, Shaanxi, China.; 6 Ultrasound Diagnostic and Treatment Center, Xijing Hospital of digestive diseases, Air Force Medical University, Xi'an, Shaanxi, China.

**Keywords:** FA oxidation, lipid uptake, lipotoxicity, oxidative stress, cardiac hypertrophy

## Abstract

**Introduction:** The heart predominantly derives its energy from fatty acid (FA) oxidation. However, the uncoupling of lipid uptake and FA oxidation can result in abnormal cardiac lipid accumulation and lipotoxicity, particularly in the context of heart failure. CD36 is a critical mediator of FA uptake in cardiac tissue. Studies have shown that genetic deletion of CD36 can prevent the onset of cardiac hypertrophy and dysfunction in murine models of obesity and diabetes. Nevertheless, the precise role of CD36 knockdown or knockout in the development and progression of cardiac dysfunction under conditions of pressure overload remains unclear.

**Objective:** This study aims to investigate the feasibility of CD36 partially knockdown in the prevention of cardiac lipotoxicity and functional impairment in pressure overload heart.

**Methods:** Cardiac-specific CD36 totally knockout (CKO) and partially knockdown (CKD) mice were induced by genetics deletion and AAV-9 CD36 shRNA injection, respectively. Both CD36 CKO and CKD mice were subjected to transverse aortic constriction (TAC) operation to induce cardiac pressure overload. Cardiac function was measured by echocardiography. Cardiac lipid accumulation, FA oxidation and metabolic sate were also examined.

**Results:** TAC operation induced significant cardiac dysfunction and pathological cardiac remodeling, accompanied by aberrant intra-myocardial lipid deposition and impaired FAO capacity. CD36 CKO attenuated aberrant lipid accumulation in the failing heart, while aggravated TAC-induced cardiac energy deprivation and oxidative stress. In contrast, CD36 CKD ameliorated TAC-induced lipid accumulation and excessive oxidative stress in the mice heart, accompanied by improved mitochondrial respiration function. Moreover, CD36 CKD induced a robust increase in glycolytic flux into the TCA cycle, which led to preserved ATP generation. As a result, CD36 CKD prevented the development of pressure overload-induced cardiac hypertrophy and dysfunction.

**Conclusion:** In this study, we reported that CD36 CKD, not CD36 CKO, was able to protect against cardiac functional impairment in the pressure-overload heart. Manipulating CD36 was a feasible strategy to achieve an optimal point which maintain cardiac energy supply while avoiding lipotoxicity.

## Introduction

The working mammalian hearts requires a substantial amount of energy to maintain contractile function and blood flow. To achieve this, ATP must be continuously generated by oxidative phosphorylation in the mitochondria, followed by glycolysis to a lesser extent 1. In the adult heart, more than 70% of substrates utilized for ATP production are derived from fatty acids (FAs), with glucose, lactate, and ketones being the remainder^2^. Depending on the nutrient conditions, the adult heart has the ability to switch between different substrates, which enable the heart to adapt to environmental change and fulfill the substantial energy requirement by adjusting metabolic functioning^3^,^4^.

It is widely accepted that the heart failure (HF) is associated with a metabolic pattern shifting from FA oxidation to glucose metabolism, which is considered adaptive to diminished oxygen consumption in the failing heart^5^. Studies conducted in animals' model of HF showed that impaired capacity for mitochondrial FAs oxidation and increased reliance on glycolysis^6^. However, this shift of metabolic substrate preference was always accompanied by decreased ATP generation. In the end-stage of HF, myocardial ATP decreased below 60-70% of its normal value^7^,^8^. Another detrimental consequence of this metabolic shift is the relative uncoupling between lipid uptake and lipid oxidation in the failing heart, leading to aberrant cardiac lipid accumulation^9^. A key study conducted by Sharma *et al.* described a subgroup of patients with contractility dysfunction showed severe cardiac metabolic dysregulation characterized by accumulation of intramyocardial triacylglycerol and lipotoxicity-related transcriptional profile change^10^. Further evidence from samples of patients undergoing left ventricular assist device placement and heart transplantation showed impaired FA oxidation and accumulation of toxic lipid accumulation^11^. These studies demonstrated a close link between the aberrant lipid accumulation and cardiac dysfunction in the progression of HF. It may seem feasible that limiting the accumulation of toxic lipid will beneficial to cardiac function during the pathogenesis of HF.

CD36, also known as FA translocase (FAT), plays a major role in FAs uptake in the heart^12^. It was reported that cardiac specific CD36 knockout mice displays significantly reduced cardiac FAs uptake and oxidation, accompanied by markedly enhanced glucose usage^13^. Studies also shown that genetic deletion of CD36 prevented the development of cardiac hypertrophy and dysfunction induced by obesity and diabetes^14^. Moreover, whole body CD36 knockout mice were protected from diet-induced cardiac dysfunction following transverse aortic constriction^15^. The cardiac protection effect of CD36 ablation in these cases was attributed to the limiting of excessive FAs uptake and subsequent prevention of toxic lipid accumulation^16^. However, another study showed that cardiomyocyte-specific CD36 deletion aggravated cardiac contractile dysfunction under the condition of pressure overload, while feeding these mice with medium-chain FAs protected these mice from developing cardiomyopathy^17^. These studies suggested that the level of CD36 expression should be maintained in a suitable extent. Total deletion of CD36 would lead to energy insufficiency, whereas over-activated CD36 would induce excessive FAs uptake and lipotoxicity. Although that the cardiac effect of CD36 deletion has been extensively studied under the condition of pressure overload, it is currently unclear the exact role of CD36 knockdown (CKD), but not knockout (CKO), plays in the development and/or progression of cardiac lipotoxicity and energy metabolism in the pressure overload heart.

In this study, we demonstrated that CD36 CKD, but not CKO, in mice heart, prevented the development of pressure overload-induced cardiac hypertrophy and dysfunction. CD36 CKD ameliorated pressure overload-induced lipid accumulation and excessive oxidative stress in the mice heart, accompanied by improved mitochondrial respiration function. Moreover, CD36 CKD induced a robust increase in glycolytic flux into the TCA cycle, which led to preserved ATP generation in the heart. By manipulating CD36, our data provide a feasible strategy to cope with both myocardial ATP decline and toxic lipid accumulation in the failing heart.

## Methods

### Animals

Eight-week-old male wild-type C57BL/6J mice were purchased from the Shanghai Biomodel Organism Science & Technology Development Lab. All the mice were housed in a temperature, humidity, and light-controlled room and allowed free access to water and food. Male mice were randomly assigned into sham or TAC groups at 10 weeks of age. All animal experimental procedures were approved by the Fourth Military Medical University Animal Use and Care Committee (No. 20230850).

### Cardiac-specific CD36 knockout mice

CD36^flox/flox^ and αMHC-MerCreMer mice (C57BL/6J background) were generated by Shanghai Model Organisms Center, Inc via the CRISPR/Cas9 technology. Conditional cardiac-specific CD36 knockout mice (CD36^flox/flox^αMHCCre+, defined as CD36 CKO mice) were generated by crossing CD36^flox/flox^ mice with αMHC-MerCreMer mice, and littermate CD36^flox/flox^ mice were used as control ones. Tamoxifen was dissolved in corn oil (20 mg/ml) and intraperitoneally injected (75 mg/kg body weight) for 5 consecutive days to induce CD36 knockout at indicated time point.

### AAV9 injection

AAV9-sh-CD36 and AAV9-NC were constructed by Hanbio Biotechnology Ltd (Shanghai, China). Cardiac-specific CD36 knockdown mice were generated by intramyocardial injection. AAV9 CD36 was injected into mouse ventricle muscular wall (three sites around the heart) when the mice were 8-weeks-old.

### Echocardiography

Echocardiography of the left ventricle was performed using a VEVO 3100 echocardiography system (Visual Sonics Inc., Toronto, Canada). Real-time ECG monitoring equipment was used to detect mouse heart rate while performing the experiment. Mice were anesthetized with 2% isoflurane, maintained under anesthesia with 1.5% isoflurane. Left ventricular fractional short-ending (LVFS) and ejection fraction (EF) were calculated from the M-mode images using computer algorithms. Diastolic trans-mitral blood flow velocities for peak early (E) and late (A) fillings was assessed by Doppler echocardiography.

### Histology

Mice hearts were fixed in 4% paraformaldehyde (PH 7.4) over night. Then, mice hearts were embedded in paraffin. Hematoxylin and eosin staining was conducted following standard procedures as previously described. Masson trichrome staining was used to detect intramyocardial collagen content. Wheat germ agglutinin was used to detect the mean cross-sectional area of cardiomyocytes.

### Western-blot analysis

Mice heart tissue was lysed with RIPA buffer containing protease inhibitor cocktail. Western blotting analysis were performed as previously described. The primary antibody against the following proteins were used: GAPDH (Proteintech, #10494-1-AP) and CD36 (Abcam, #ab252923).

### Transmission electron microscopy (TEM)

Left ventricular tissue was dissected into 3-mm^3^ pieces and fixed overnight in 2.5% glutaraldehyde. Images were obtained using a TEM (JEM-1230, JEOL Ltd., Japan) at 300kV and analyzed using ImageJ software.

### Dihydroethidium (DHE) staining

Intracellular superoxide anion (O2•-) levels in the mice hearts tissue were detected by DHE staining. Images were obtained with a confocal laser-scanning microscope (Nikon A1R MP+ Confocal Microscope, Nikon, Japan). The images were analyzed with ImagePro Plus image analysis software.

### Measurement of MDA level in heart tissue

Lipid Peroxidation MDA Assay Kits (S0131, Beyotime Biotechnology, Jiangsu, China) was using to detect MDA levels in mice heart tissue, according to protocols provided by the manufacturer.

### Lipidomic analysis

The heart samples were stored at -80°C until metabolite extraction, and lipodomic profiling was conducted using ultrahigh-performance liquid chromatography/tandem mass spectrometry (LC/MS).

### Isolation of adult cardiomyocytes

The mouse heart was digested with collagenase II, and dissociated cells were sedimented by gravity. Cardiomyocytes were isolated, then were fixed in 4% paraformaldehyde and stained for subsequent analysis.

### Fluorescent imaging of lipid droplets (LDs)

Bodipy493/503(Invitrogen, #D2191) was used to stain lipid droplets in cardiomyocytes. The images were obtained by Nikon A1 plus confocal laser-scanning microscope (Nikon, Japan).

### Measurement of mitochondrial function

Mitochondrial function was estimated as oxygen consumption using high-resolution respirometry (Oxygraph-2k, Oroboros Instruments).

### Metabolomics

For U-^13^C-labeled glucose flux analyses, after a 6 h fast, mice were intraperitoneal injected with ^13^C6-glucose. One hour after injection, mice heart was isolated and content of metabolites were determined.

### Statistical analysis

All values were presented as Mean ± Standard Error (Mean ± SEM). Difference two groups was assessed with two tailed Students' t-test. For four groups, the data were subjected to One-way ANOVA. A value of P < 0.05 was considered as statistically significant difference.

## Results

### Excessive lipid accumulation was observed in the pressure overload heart

**Figure 1:**
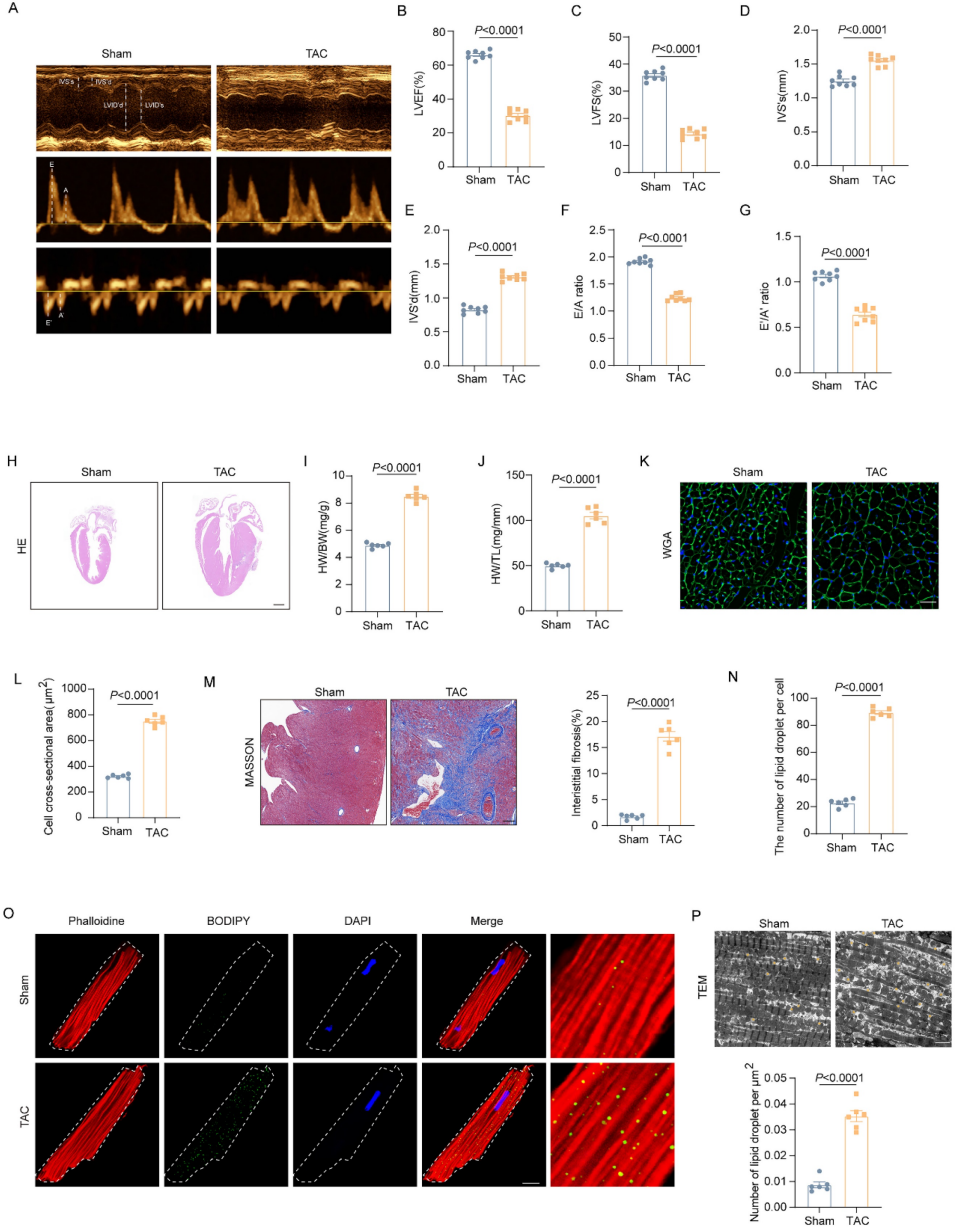
** Excessive lipid accumulation was observed in the pressure overload heart. (A-G)** Representative images of cardiac function and quantitative analysis (LVEF, LVFS, IVS's, IVS'd, E/A ratio and e'/a' ration) **(H)** Representative images of hematoxylin and eosin staining of the mice hearts. Scale bar, 2 mm. **(I)** Ratio of heart weight to body weight. **(G)** Ratio of heart weight to tibia length. **(K)** Representative images of wheat germ agglutinin (WGA) staining. **(L)** Quantitative analysis of cross-sectional cell area, Scale bar, 20 μm. **(M)** Representative Masson trichrome staining staining images and quantitative analysis of cell area, Scale bar, 20 μm. **(N-O)** Accumulation of neutral lipid droplets (vivid green dots) in the isolated adult cardiomyocytes was detected by Bodipy 493/503 staining and the number of lipid droplets per cell was analyzed. Scale bar, 2 μm. **(P)** Representative transmission electron microscopic images of lipid droplets and number of lipid droplet per μm^2^. Data are presented as mean ± SEM; Statistical Analysis based on the Student's t-test.

To explore the role of dysregulated lipid metabolism in the development of HF, we used the TAC mouse model to induce LV pressure overload that causes cardiac hypertrophy and HF. As shown in figure 1A-G, the echocardiographic results showed that wild-type (WT) mice exhibited significantly impaired cardiac systolic and diastolic function 4 weeks after TAC operation, as indicated by decreased EF, FS, E/A ratio and e'/a' ratio. Moreover, TAC induced pronounced cardiac hypertrophy and cardiac fibrosis in the hearts of WT mice. As shown in Figure 1H-M, when compared with those mice in the Sham group, TAC mice had significantly increased interventricular septum (IVS), enlarged heart size and cardiomyocytes sectional area (CSA), and elevated ratio of heart weight to body weight or tibia length. To investigate the involvement of dysregulated lipid metabolism in the development of pressure overload induced HF, cardiac lipid accumulation was detected by fluorescence staining and TEM. The fluorescence staining results of isolated adult cardiomyocytes showed that aberrant LDs accumulation in the heart of mice underwent TAC (Figure 1N-O). Meanwhile, TEM results further confirmed the LDs deposition (Figure 1P). These data suggested that excessive lipid accumulation was involved in the development of HF.

### CD36 CKO attenuated aberrant lipid accumulation while exacerbated lipid overload-induced cardiac dysfunction in the TAC heart

**Figure 2:**
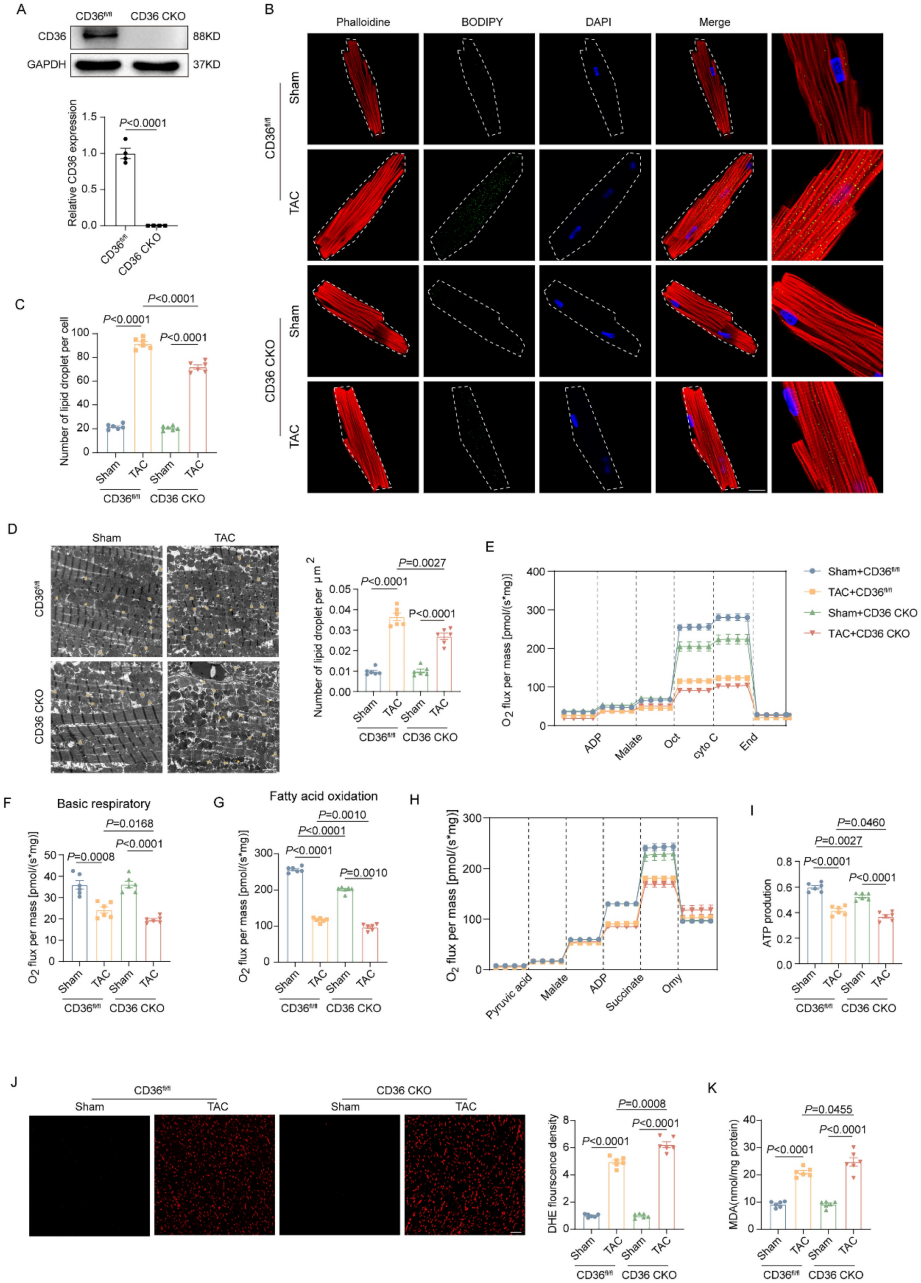
** CD36 CKO attenuated aberrant lipid accumulation in the TAC heart, but aggravated TAC-induced cardiac energy deprivation and oxidative stress. (A)** Representative western blots of CD36 and quantitative analysis. **(B-C)** Accumulation of neutral lipid droplets (vivid green dots) in the isolated adult cardiomyocytes was detected by Bodipy 493/503 staining and the number of lipid droplets per cell was analyzed. **(D)** Representative transmission electron microscopic images of lipid droplets and number of lipid droplet per μm^2^. **(E)** Representative experiment to detect FA oxidation capacity using the O2K instrument. **(F)** Basic respiratory measured by oxygen consumption. **(G)** FAO measured by oxygen consumption. **(H)** Representative experiment to detect ATP production capacity using the O2K instrument. **(I)** ATP production. **(J)** Representative images of DHE staining and quantitative analysis. **(K)** MDA content in heart tissue. Data are presented as mean ± SEM; Statistical Analysis based on the One-way ANOVA. n=8 animals in every group.

Given the critical role of CD36 in cardiac FAs uptake, cardiac-specific CD36 knockout mice was constructed and subject to TAC. The CD36 expression in the CKO heart was almost absent (Figure 2A). In mice subjected to Sham operation, both control mice (CD36^fl/fl^) and CD36 CKO mice showed limited amounts of LDs in the myocardium. As expected, CD36 CKO attenuated TAC-induced cardiac lipid accumulation, as evidenced by significantly reduced intramyocardial LDs number in TAC-treated CD36 CKO mice when compared with those mice underwent Sham treatment (Figure 2B-D). These data suggested that CD36 deletion was able to prevent TAC-induced aberrant lipid accumulation. Considering the critical role of lipotoxicity in TAC-induced cardiac dysfunction, cardiac function was also determined by echocardiography (Figure S1). Similar with the observation in the WT mice, TAC induced significant cardiac dysfunction and cardiac hypertrophy in CD36^fl/fl^ mice, as indicate by decreased LVEF, LVFS, E/A and e'/a' as well as increased IVS. However, CD36 CKO further decreased indices in both cardiac systolic and diastolic function, suggesting that CD36 CKO aggravated TAC-induced cardiac dysfunction.

### CD36 CKO aggravated TAC-induced cardiac energy deprivation and oxidative stress

We further investigate the potential influence of CD36 CKO in the mitochondrial function and oxidative stress. As shown in Figure 2E-I, mitochondrial respiration function was significantly impaired in the TAC-heart, as indicated by decreased basal respiration and FAO capacity. Meanwhile, the ATP production in the TAC heart was reduced by approximately 30% when compared with Sham heart. Interestingly, CD36 CKO heart showed comparable basal respiratory capacity but decreased FAO capacity when compared with control mice with Sham operation. However, in mice subjected to TAC operation, TAC-CD36 CKO heart exhibited significantly decreased level in both basal respiratory and FAO oxidation capacity when compared with TAC-CD36^fl/fl^ mice. Meanwhile, the TAC-CD36 CKO heart showed further reduction in ATP generation compared with TAC-control heart. These data suggested that CD36 CKO aggravated TAC-induced energy deprivation by inhibiting mitochondrial energy metabolism. Incomplete FAs oxidation was closely related to cellular oxidative stress during the development of HF. Therefore, we wonder if CD36 CKO was able to prevent TAC-induced oxidative stress. The oxidative level was evidently increased in the TAC heart, as evidenced by increased DHE and MDA level (Figure 2J-K). However, CD36 CKO failed in attenuating oxidative stress. In contrast, CD36 CKO in the TAC heart exacerbated oxidative stress, as indicated by further increased DHE and MDA level (Figure 2J-K). Taken together, these data suggested that although CD36 CKO was able to prevent cardiac lipid accumulation, it aggravated cardiac energy deprivation and oxidative stress in the TAC heart.

### CD36 CKD ameliorated TAC-induced cardiac dysfunction and cardiac hypertrophy

Having seen that CD36 CKO aggravated cardiac energy deprivation and oxidative stress in the TAC-heart, we proposed that CD36 knockdown might be a feasible strategy to prevent lipid accumulation and simultaneously preserved energy production in failing heart. Hence, recombinant adeno-associated virus encoding CD36 shRNA (AAV9-sh-CD36) was constructed and intramyocardial injected into mice heart to knockdown CD36.

**Figure 3:**
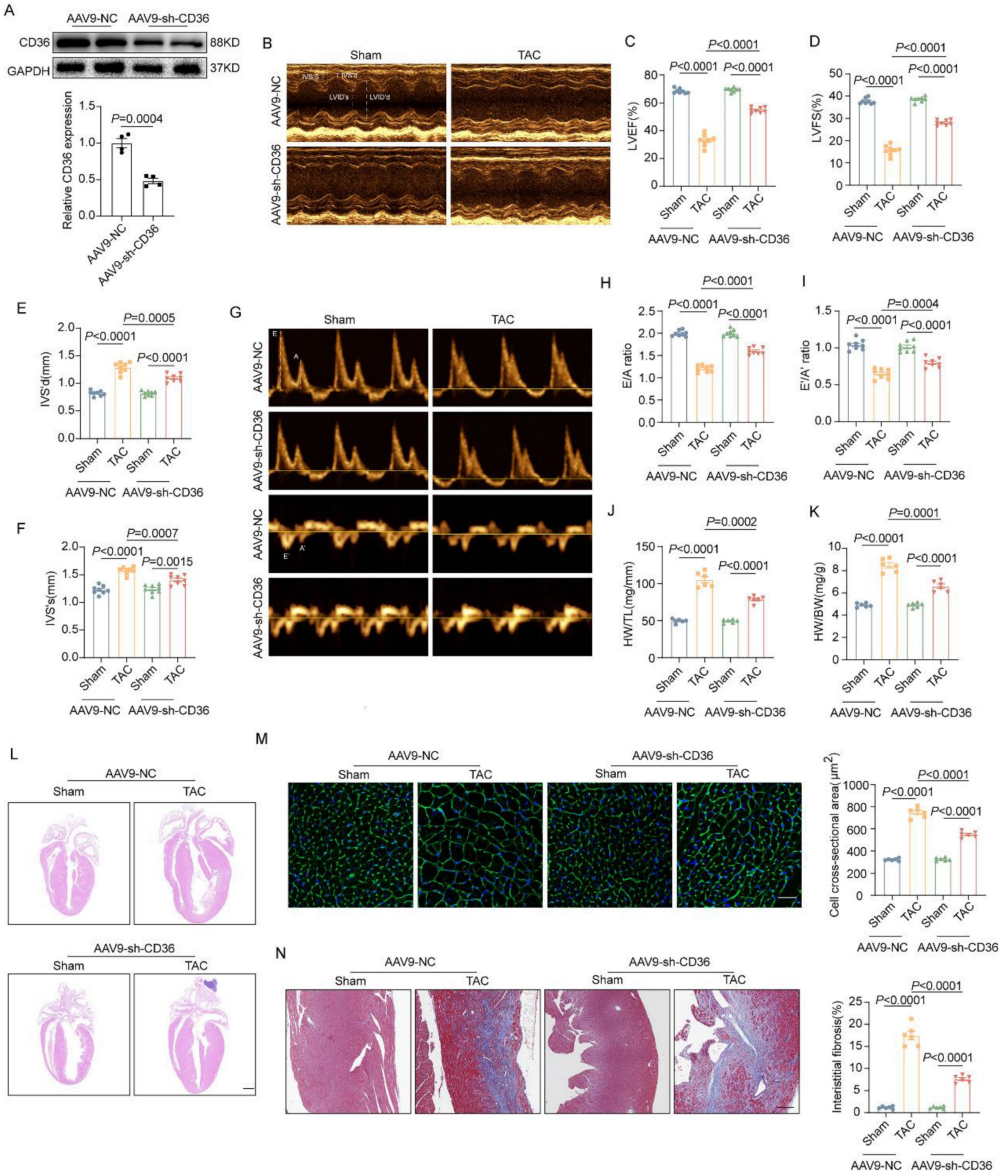
** CD36 CKD ameliorated TAC-induced cardiac dysfunction and cardiac hypertrophy. (A)**: Representative western blots of CD36 and quantitative analysis. **(B-F)** Representative images and quantitative analysis (LVEF, LVFS, IVS's and IVS'd) of M-mode echocardiography. **(G-I)** Representative Doppler flow measurement of mitral inflow and quantitative analysis of E/A ratio and e'/a' ratio. **(J)** Heart weight to body weight ratio. **(K)** Heart weight to tibia length ratio. **(L)** Representative images of hematoxylin and eosin staining of the mice hearts. Scale bar, 2 mm. **(M)** Representative wheat germ agglutinin (WGA) staining images and quantitative analysis of cross-sectional cell area, Scale bar, 20 μm. **(N)** Representative Masson trichrome staining images and quantitative analysis of interstitial fibrosis area, Scale bar, 20 μm. Data are presented as mean ± SEM; Statistical Analysis based on the One-way ANOVA. n=8 animals in every group.

As shown in Figure 3A, AAV9-sh-CD36 injection reduced CD36 expression by ~50%. Under basal condition, CD36 CKD showed no significant alteration in cardiac function and cardiac hypertrophy in Sham-heart, as indicated by comparable EF, FS, E/A ratio, e'/a' ratio and indicator of gross morphology of hearts (Figure 3B-F). However, unlike the results observed in the CD36 CKO heart, CD36 CKD ameliorated cardiac dysfunction and hypertrophy in the pressure overload heart. As shown in Figure 3B-I, TAC-CD36 CKD heart showed significantly higher EF, FS, E/A ratio and e'/a' ratio when compared with TAC hearts injected with AAV9-NC. Meanwhile, CD36 CKD prevented the development of cardiac hypertrophy and cardiac fibrosis, as indicated by smaller heart size, decreased CSA, reduced heart weight/ tibia length and less fibrotic area (Figure 3J-N). These data suggested that CD36 CKD, not CD36 CKO was able to protect against TAC-induced cardiac hypertrophy and functional impairment.

### CD36 CKD prevented TAC-induced toxic lipid accumulation

**Figure 4:**
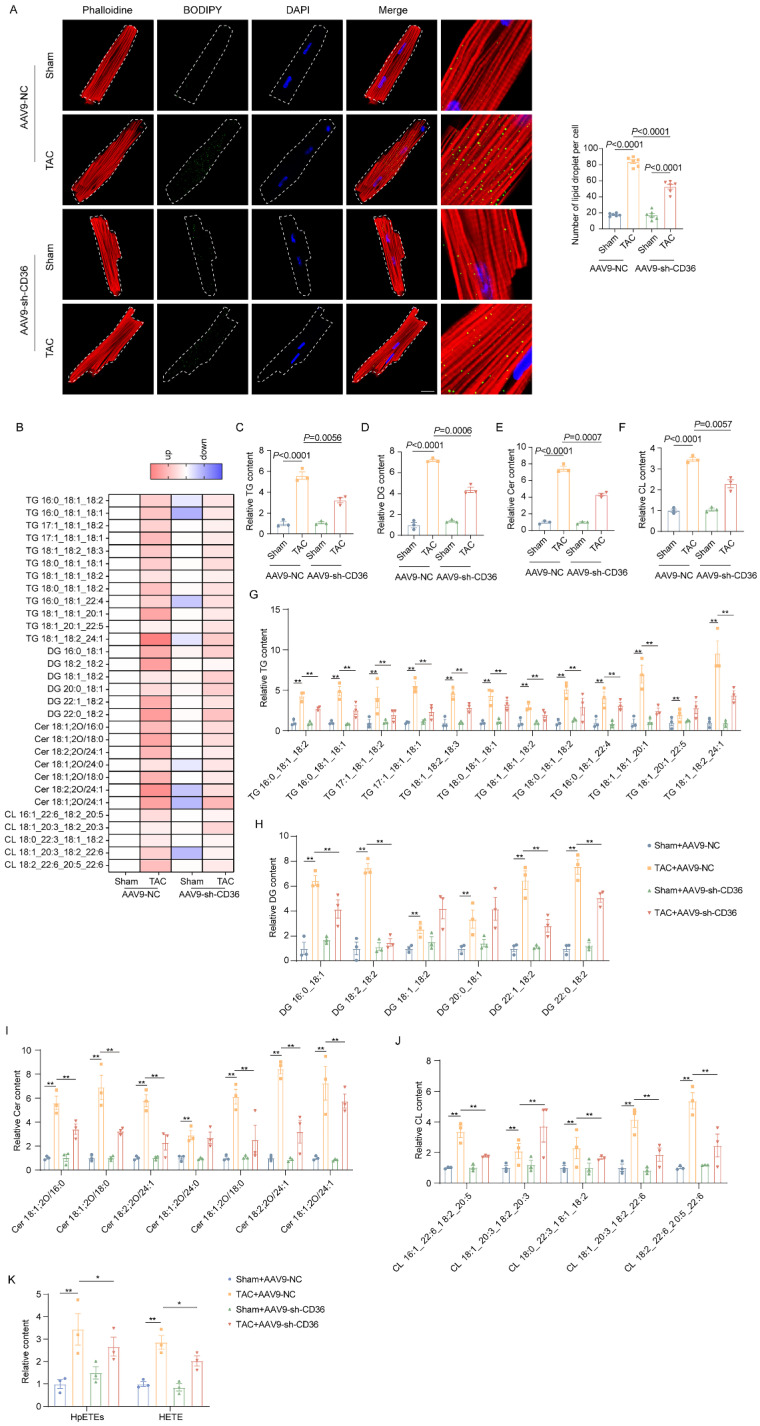
** CD36 CKD prevented TAC-induced toxic lipid accumulation. (A)** Accumulation of neutral lipid droplets (vivid green dots) in the isolated adult cardiomyocytes was detected by Bodipy 493/503 staining and the number of lipid droplets per cell was analyzed. Scale bar, 2 μm. **(B)** Heatmap of the subclasses of lipid level in the heart; n = 4 per group. **(C)** Relative triglyceride (TAG) concentration in the heart. **(D)** Relative diglyceride (DAG) concentration in the heart. **(E)** Relative ceramide (Cer) concentration in the heart. **(F)** Relative cholesterol (Chol) content in the heart. **(G)** Relative major Triglycerides TAG) species content in mice heart. **(H)** Relative major diglyceride (DAG) species content in mice heart. **(I)** Relative major ceramide (Cer) species content in mice heart. **(J)** Relative major cholesterol (Chol) species content in mice heart. **(K)** The content of oxidatively modified lipids in the heart of mice in the indicated groups. Data are presented as mean ± SEM; Statistical Analysis based on the One-way ANOVA. n=4-8 animals in every group.

We further investigate the influence of CD36 CKD in cardiac lipid accumulation under normal or pressure overload condition. As shown in Figure 4A, CD36 CKD also did not influence the LDs number in the Sham heart. Similar to the results observed in the CD36 CKO heart, CD36 CKD prevented the lipid accumulation in the pressure overload heart, as indicated by significantly decreased intramyocardial LDs numbers (Figure 4A). Intramyocardial lipid content was further analyzed using lipidomics. Triglycerides (TAG) is the predominant form of lipid storage, whereas diglycerides (DAG) and ceramides are the most thoroughly causes of lipotoxicity. Therefore, we quantified TAG, DAG, cholesterol, and ceramides in mice. As shown in Figure 4B-F, cardiac TAG, DAG and ceramides level was significantly increased in the TAC-AAV9-NC heart when compared with Sham-AAV9-NC heart. In contrast, AAV9-sh-CD36 injection ameliorated the accumulation of TAG (Figure 4C&G). Of note, DAG and ceramides, which were widely involved in oxidative stress and apoptosis, was also evidently decreased by AAV9-sh-CD36 injection in the TAC hearts (Figure 4D-E&H-J). Moreover, oxidatively modified lipids including HpETEs and HETE were decreased in the CD36 CKD heart compared with TAC-AAV9-NC heart (Figure 4K). These data suggested that CD36 CKD was able to prevent the toxic lipid deposition in the failing heart, thereby attenuating lipotoxicity.

### CD36 CKD rescued energy production and attenuated oxidative stress by improving mitochondrial function

**Figure 5:**
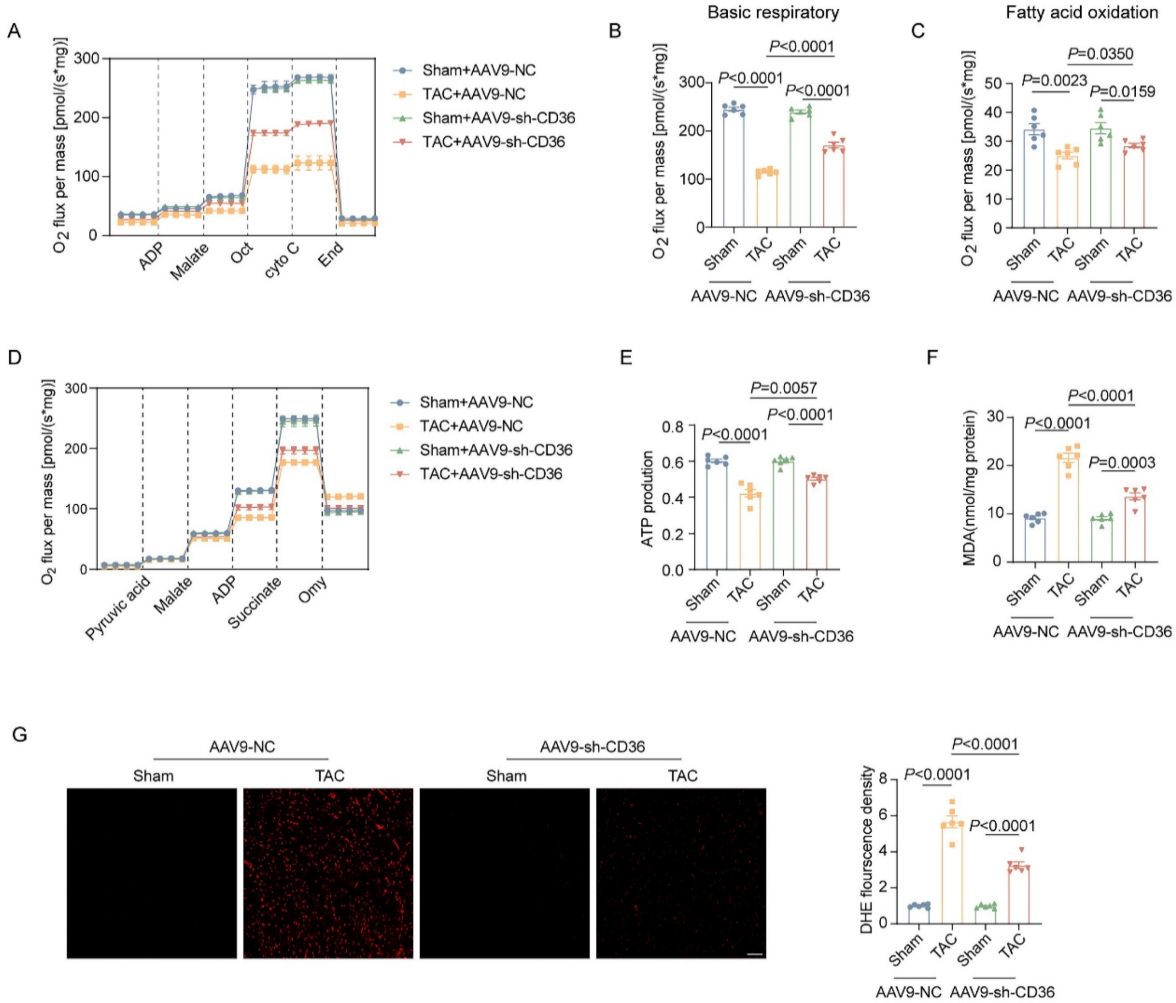
** CD36 CKD rescued energy production and attenuated oxidative stress by improving mitochondrial function. (A)** Representative experiment to detect FA oxidation capacity using the O2K instrument. **(B)** Basic respiratory measured by oxygen consumption. **(C)** FAO measured by oxygen consumption. **(D)** Representative experiment to detect ATP production capacity using the O2K instrument. **(E)** ATP production. **(F)** MDA content in heart tissue. **(G)** Representative images of DHE staining and quantitative analysis. Data are presented as mean ± SEM; Statistical Analysis based on the One-way ANOVA. n=8 animals in every group.

Mitochondrial respiratory function was also determined by O2K instruments. As shown in Figure 5A-C, in Sham mice, CD36 CKD showed no significant change in both basal respiration and FA oxidation, as well as cardiac ATP content (Figure 5D-E). In contrast, CD36 CKD improved mitochondrial respiratory function in TAC mice, as evidenced by increased basal respiration (Figure 5B). Moreover, the ATP content in the TAC heart was also elevated by ~25% in AAV9-sh CD36 injected hearts when compared with those injected with AAV9 NC (Figure 5E). Mitochondrial oxidative phosphorylation is a major source of oxidative stress. As shown in Figure 5F-G, CD36 CKD did not affect the oxidative stress level in the Sham heart, as comparable DHE density and MDA level. Moreover, CD36 CKD attenuated TAC induced cardiac oxidative stress, as indicated by reduced MDA content and DHE fluorescence density. These data suggested that CD36 CKD rescued energy production and attenuated oxidative stress in the failing heart, which was associated with improved mitochondrial function.

### CD36 CKD enhanced glycolytic flux into the TCA cycle to preserve ATP production

**Figure 6:**
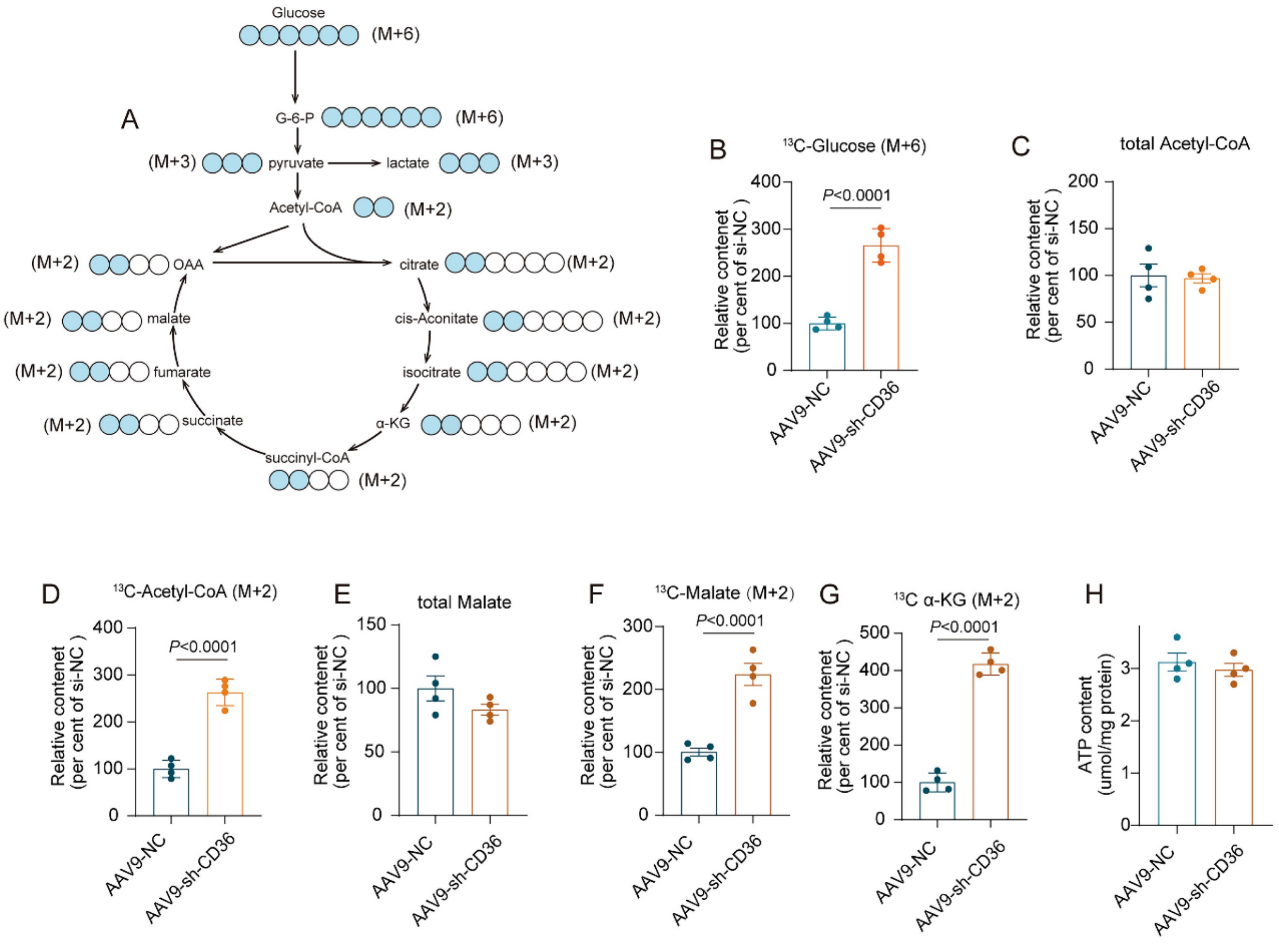
** CD36 CKD enhanced glycolytic flux into the TCA cycle to preserve ATP production. (A)** Metabolic profiling in glucose oxidation pathway. **(B)-(G)** Metabolic flow study with ^13^C_6_-glucose. Total metabolites and 13C-labeled metabolites were quantified. **(H)** ATP content.

To investigate whether glucose metabolism was involved in energetic rescue in the CD36 CKD heart, uptake and metabolism of ^13^C-labeled glucose was determined by metabolic flow analysis. After a 6 h fast, both Control mice and CD36 CKD mice were intraperitoneal injected with ^13^C-glucose. One hour after injection, mice heart was isolated and content of metabolites were determined. The graphical illustration of glucose oxidation was shown in figure 6A. CD36 knockdown significantly increased the uptake of glucose, as indicated by elevated ^13^C-labeled glucose content (Figure 6B). However, the relative content of total acetyl-CoA and malate, which were important intermediate product of TCA cycle, showed no significant change in the CD36CKD heart (Figure 6C&E). Meanwhile, the ATP production also showed no change (Figure 6H). In contrast, ^13^C-labeled intermediates of TCA cycle including acetyl-CoA, malate and α-KG was evidently increased by CD 36 knockdown (Figure 6D-G). These data suggested that the increased glycolytic into the TCA cycle was able to partially compensate the FAO inhibition to preserve ATP production in the CD36 CKD heart.

## Discussion

Serval studies has reported FA oxidation capacity impairment in the failing heart, leading to imbalance FAs uptake and FAs degradation^18^,^19^. The aberrant intramyocardial lipid accumulation and related lipotoxicity was a hallmark of the failing heart, as well as myocardium of patients with obesity and diabetes millets^1^,^20^,^21^. Hence, disruption of FAs uptake was considered as a potential feasible strategy for the treatment of cardiac lipotoxicity during the process of multiple cardiometabolic diseases. However, Yogi *et al.* reported that cardiac specific CD36 deletion exacerbated cardiac hypertrophy and functional impairment under pressure overload condition^17^. Further results demonstrated that the detrimental effect of CD36 knockout on pressure overload heart was attributed to the insufficient energy supply, suggesting that CD36-dependent FAs uptake was indispensable for the sufficient energy production to maintain the cardiomyocytes contraction in the context of pressure overload^17^. Considering the predominate role of FAs oxidation in the energy supply for myocardium, it is intelligible that blocking the major of cardiac fuel would be deleterious. Although studies found that CD36 inhibition led to a significant activation of glucose metabolism flux into the TCA cycle, it was not sufficient to compensate for ATP loss in the failing heart. These data suggested that long chain FA taken up through CD36 are central energy substrates for sufficient ATP production, thereby maintaining cardiac contractile function even under increased workload condition. Here, in this study, we reported that CD36 CKD, but not CD36 CKO was able to reach an optimal solution which both prevented the cardiac lipid accumulation and preserved FAs-derived energy production. Interestingly, feeding CD36 CKO mice a medium-chain fat-enriched diet, which bypasses CD36-dependent FAs uptake pathway, is able to protect these mice from developing HF^17^. These data suggested that preserving, but not blocking FAs uptake was a feasible strategy to maintain cardiac function in the failing heart.

Although FAs oxidation provides the primary energy for cardiomyocytes, excessive FAs accumulation would induce oxidative stress and mitochondrial damage^22^,^23^. The accumulated FAs are then incorporated into triglycerides (TAG) and phospholipids, as well as multiple other lipid subspecies^24^. The TAG is the most easily detected among all these species. However, it is not TAG, but toxic nonpolar lipids, DAGs and ceramides, are responsible for the impaired cardiac function^2^. The relevance of our finding is supported by the notion that overexpression of DGAT1 (converts the toxic DAG to TAG) reversed cardiac dysfunction in hearts of lipotoxic models despite increased TAG accumulation^25^,^26^. In contrast, DAGs and ceramides are considered to be strongly associated with insulin resistance and predictors for poor prognosis in patients with cardiovascular diseases^27^-^29^. In this study, we found that TAG, DAG and ceramides were all significantly increased in the failing heart of pressure overload mice, accompanied by evidently increased oxidative stress level and mitochondrial functional impairment. Moreover, CD36 CKD attenuated the accumulation of both polar and nonpolar lipids, which could be also responsible for the protective effect of CD36 CKD in mitochondrial function and cardiac function. In reverse, defective mitochondrial function and subsequent incomplete FAs oxidation could lead to the accumulation of medium chain acyl carnitines, which was also toxic. Hence, there might be vicious cycle in the failing heart which was consisted between the impaired mitochondrial function and toxic lipid accumulation. Inhibition of FAs uptake by CD36 CKD blocked the initiation of the vicious cycle, thereby preventing the development of lipotoxicity and cardiac dysfunction in the failing heart.

The relative increase of glucose flux into the TCA cycle is another feature of metabolic reprogramming in the failing heart^3^. The shift towards glucose oxidation in the failing heart was considered to be beneficial given its higher energetic efficiency than that of FAs oxidation^30^. Therefore, stimulation of glucose oxidation might be a therapeutic strategy to improve cardiac energy supply and cardiac efficiency in the failing heart. In this study, we found that inhibition of CD36 further increased the glucose flux into TCA cycle in the failing heart, thereby preserving the ATP production and protecting the cardiac function. Consistent with our observation, previous study found that inhibition of carnitine-Opalmitoyltransferase 1 (CPT1) improved cardiac energetics, exercise capacity and clinical symptoms in patients with dilated or hypertrophic cardiomyopathy^31^. However, it was also reported that the increase in glucose uptake and glycolytic flux was not accompanied by a relative increase in glucose oxidation. This uncoupling between the glucose uptake and oxidation was possibly attributed to a mismatch between increased activity of glycolytic enzyme and unchanged or even decreased mitochondrial pyruvate oxidation^32^. The glucose metabolic pathway seems to be far more than acting as a source for intracellular ATP in the myocardium. The intermediate metabolites of glucose oxidation was widely involved in cell proliferation and hypertrophy^33^. For instance, recent study showed that alpha-ketoglutarate, an intermediate of glucose oxidation, was able to promote transcription of hypertrophy-related genes in cardiomyocytes via regulation of histone methylation^34^. In addition, experimental results from metabolome analysis also revealed that the increased glycolytic flux was more inclined to synthesize biomaterials for structural remodeling rather than ATP production in the CD36 CKO heart^35^. Taken together, it is still controversy that the precise meaning of the increased glycolytic flux in the pathogenesis of HF, further studies are needed to look into that.

Given the important role of excessive ROS production in pathological cardiac remodeling and the development of HF, various attempts have been made to decrease ROS production or scavenge ROS in the cardiomyocytes^36^. In cardiomyocytes, ROS are generated primarily by mitochondrial oxidative phosphorylation, ADPH oxidase, xanthine oxidase, and uncoupled nitric oxide synthase^37^. An imbalance between ROS production and ROS scavenging by antioxidant defense mechanisms was an important initiator in the development of HF^36^. Of note, oxidative free radicals can act on the lipid to damage biological membranes and produce cytotoxic peroxidation products^38^. As organelles with a double-layer membrane structure, mitochondria are sensitive to ROS-induced damage. Here, we found that both mitochondrial basal respiratory and FAs oxidation were significantly impaired in the failing heart, which could also be attributed to ROS associated mitochondrial membrane damage. The unsaturated FAs can be transformed into lipid hydroperoxides and other lipid peroxidation products^39^. Therefore, there could be a positive correlation between the amount of unsaturated lipids and lipid peroxidation products formed in the cardiomyocytes. Based on this information, impeding the uptake of FAs into the cardiomyocytes could be a viable option to preventing the generation of lipid peroxidation products. Here, in this study, by inhibiting the CD36-mediated FAs uptake, we providing an effective method to decrease the cellular oxidative stress level and improve mitochondrial respiratory function.

There is still limitation in our study for identifying the exact portion of FAs-derived ATP production and glucose-derived ATP generation in CD36 CKD or CKO hearts. Further studies are necessary to get precise measurement of the metabolic remodeling induced by CD36 CKD and CKO. Besides, a comparative study with CD36 overexpression is required to elucidate the specific effect of CD36 in cardiac lipotoxicity and lipid peroxidation production.

In conclusion, we found that cardiac-specific CD36 CKD prevented excessive lipid accumulation and lipotoxicity in the pressure overload heart. Moreover, CD36 CKO induced an enhanced glycolytic flux into the TCA cycle, thereby preserving ATP production. Hence, manipulating CD36 might be a feasible therapeutic strategy to regulating metabolic disorder in the failing heart.

## Supplementary Material

Supplementary figure.
