# Supplementary material for: CD36 knockdown attenuates pressure overload-induced cardiac injury by preventing lipotoxicity and improving myocardial energy metabolism
Source: Int J Med Sci. 2025 Feb 18;22(5):1223–36. doi: 10.7150/ijms.107224 (PMC11866539; doi:10.7150/ijms.107224)
Supplement: Supplementary file 1 — Supplementary figure. [file ijmsv22p1223s1.pdf]

Supplementary figures

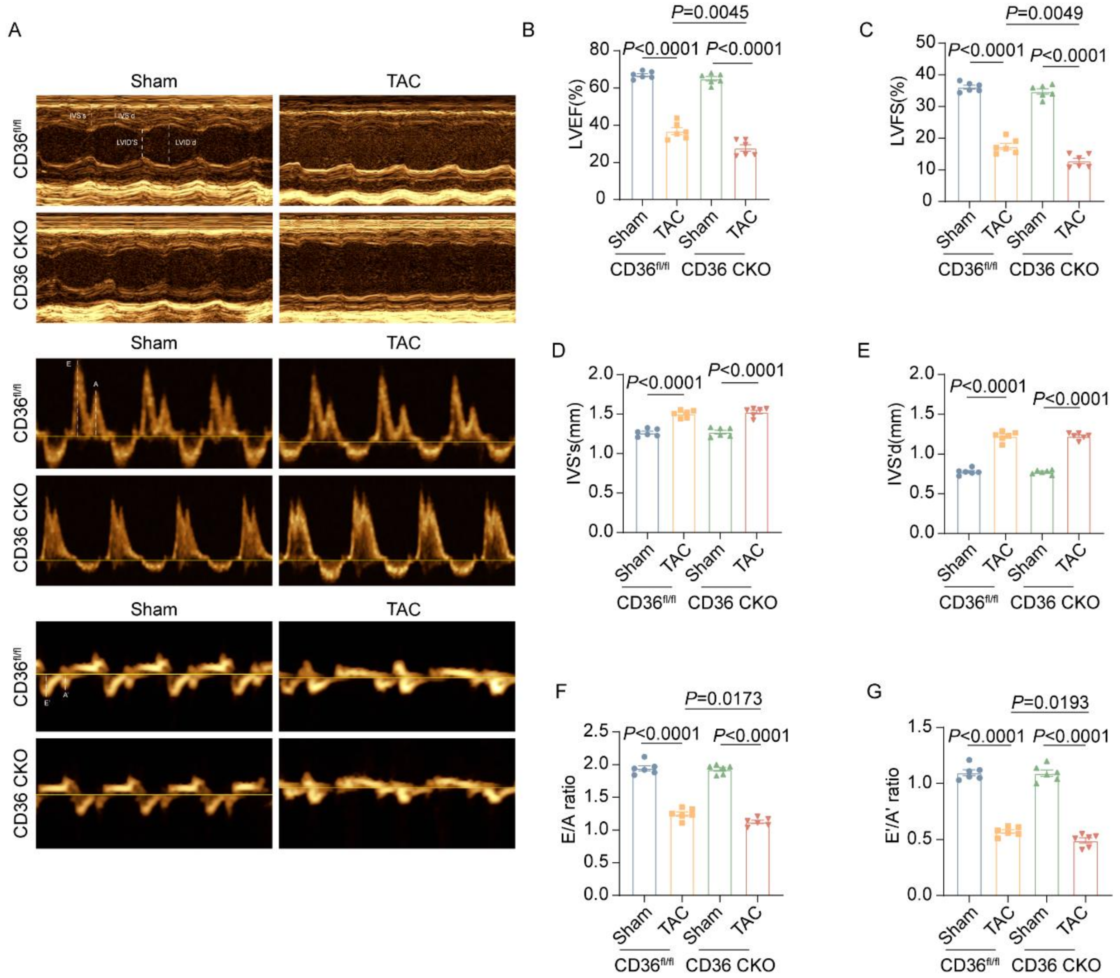

S1: CD36 CKO aggravated TAC-induced cardiac dysfunction.

(A) Representative images of cardiac function.

(B-E) Representative quantitative analysis (LVEF, LVFS, IVS's and IVS'd) of M-mode echocardiography.

(F-G) Representative Doppler flow measurement of mitral inflow and quantitative analysis of E/A ratio and E'/A' ratio.
